# Jiangzhi Ligan Decoction Inhibits GSDMD-Mediated Canonical/Noncanonical Pyroptosis Pathways and Alleviates High-Fat Diet-Induced Nonalcoholic Fatty Liver Disease

**DOI:** 10.1155/2021/9963534

**Published:** 2021-06-18

**Authors:** Kangkang Yin, Xiao Zhou, Wei Jiang, Linlin Wang, Ziwei Dai, Biao Tang

**Affiliations:** Medical School, Hunan University of Chinese Medicine, Changsha 410208, China

## Abstract

Increasing evidence suggests that gasdermin D (GSDMD) mediated pyroptosis signaling pathways play a vital role in the pathogenesis of nonalcoholic fatty liver disease (NAFLD). Jiangzhi Ligan Decoction (JZLGD) has been verified to prevent NAFLD, but its specific mechanism has not been determined. In this study, an NAFLD model was established in Sprague-Dawley rats by a high-fat diet (HFD). After 12 weeks, JZLGD was orally administered once a day for 6 additional weeks. We investigated the effects of JZLGD on NAFLD rats and determined the GSDMD pathway-associated proteins to explore whether such effects were associated with pyroptosis. Our data show that JZLGD significantly reduced the liver index; improved serum lipid levels, liver function parameters, and lipid droplet content; and relieved NAFLD. We further found that the serum levels of the proinflammatory factors interleukin-1*β* (IL-1*β*), IL-18, tumor necrosis factor-*α*, and IL-6 were obviously decreased in the JZLGD group. HFD rats treated with GSDMD exhibited NLRP3, caspase-1, lipopolysaccharide (LPS), and caspase-11 activation; however, these effects were blunted by JZLGD treatment. Taken together, JZLGD may exert hepatoprotective effects against NAFLD in a rat HFD model by regulating GSDMD-mediated canonical/noncanonical pyroptosis pathways.

## 1. Introduction

Nonalcoholic fatty liver disease (NAFLD) is a global health problem, and its incidence is rapidly increasing, especially in the United States, where its prevalence is 20–30% [1]. Its prevalence in Asia is 7–20% [2]. With the changes in people's diet and lifestyle, the incidence of NAFLD has gradually increased among the younger population, causing a burden on public healthcare systems. In some patients, NAFLD will progress to hepatitis, liver fibrosis, and cirrhosis, increasing the risk of hepatocellular carcinoma. However, the pathological mechanism underlying NAFLD is complex, and there is no effective drug at present [3], so it is very important to identify therapeutic targets for NAFLD treatment.

The pathogenesis of NAFLD is related to lipid metabolism disorder, lipid accumulation, insulin resistance, and inflammation [4, 5]. In particular, hepatic inflammation is an essential pathophysiological process during NAFLD [6]. Additionally, a study showed that inflammatory activation results in hepatic insulin resistance and steatohepatitis [7]. The occurrence and development of inflammation will aggravate the deterioration of NAFLD and make NAFLD progress into cirrhosis. Pyroptosis is a form of proinflammatory cell death, and gasdermin D (GSDMD) is the executive molecule [8]. Cleavage of GSDMD by caspase-1 and caspase-4/5/11 leads to translocation of the N-terminal fragment to the plasma membrane, which then forms pores (10–15 nm in diameter) that facilitate pyroptotic cell death to release interleukin-1*β* (IL-1*β*) and IL-18 [9, 10, 11]. On the one hand, the activation of caspase-1 requires the NLRP3 inflammasome, which recognizes diverse stimuli including pathogen-associated molecular patterns and endogenous damage-associated molecular patterns to activate pro-caspase-1 cleavage into the active form caspase-1 [12]. This pyroptotic process, which is mediated by NLRP3/caspase-1/GSDMD, is called the canonical pyroptosis pathway. On the other hand, the occurrence of noncanonical inflammasome-mediated pyroptosis depends on caspase-11/4/5 to recognize and bind lipopolysaccharide (LPS), in which case the compound activates GSDMD to cause noncanonical pyroptosis [13–15]. Recent studies have shown that pyroptosis mediates the death of hepatocytes and aggravates the processes of inflammation and fibrosis in the pathological development of NAFLD, which has become an important potential target for NAFLD treatment [16].

In traditional Chinese medicine (TCM) theory, the pathogenesis of NAFLD includes spleen vacuity, liver stagnation, and phlegm-damp obstruction [17]. TCM is effective in treating NAFLD, but there are few related studies. Jiangzhi Ligan Decoction (JZLGD), a classical Chinese herbal formula, is a widely used prescription in clinical practice. JZLGD is composed of six kinds of Chinese medicines, including *Alisma orientale* (Zexie), *Salvia miltiorrhiza* (Danshen), Cassis Tora (Juemingzi), Curcumae Radix (Yujin), Sargassum (Haizao), Lotus Leaves (Heye). Previous experiments showed that JZLGD regulates lipid metabolism, exerts hepatoprotective effects, and contributes to weight loss. It has been demonstrated that JZLGD plays an important role in NAFLD treatment, but the underlying mechanisms are not clear. Pyroptosis is closely related to the inflammatory response and plays an indispensable role in the development of NAFLD. Inhibition of pyroptosis in NAFLD can protect the liver from inflammation and delay the progression of liver disease; hence, we hypothesized that the beneficial effects of JZLGD on the progression of NAFLD may be related to the canonical and noncanonical pyroptosis pathways. Therefore, an NAFLD rat model was established to evaluate the effects of JZLGD on NAFLD and explore the underlying molecular mechanisms.

## 2. Materials and Methods

### 2.1. Materials

Sodium pentobarbital (57-33-0) was purchased from TCI Shanghai; lysis buffer for Western blot and immunoprecipitation (IP) analysis (P0013) was purchased from Beyotime Biotechnology. Protease inhibitor cocktail (without EDTA, 100× stock solution in DMSO) (B14002) and phosphatase inhibitor cocktail (100× stock solution) (B15002) were purchased from Bimake. The rat IL-1*β* ELISA Kit (GN-R30172), the rat IL-18 ELISA Kit (GN-R30168), the rat TNF-*α* ELISA Kit (GN-R31092), and the rat IL-6 ELISA Kit (GN-R30201) were purchased from Gaining Biological. The LPS detection kit (H178) was purchased from NanJing JianCheng Bioengineering Institute. Monoclonal mouse anti-GSDMD (SC-393581) and anti-caspase-11 (SC-374615) were purchased from Santa Cruz Biotechnology. Monoclonal rabbit anti-GSDMD-N (93709) was purchased from Cell Signaling Technology. Polyclonal rabbit anti-caspase-1 (NBP1-45433) and anti-NLRP3 (NBP2-12446) were purchased from Novus Biologicals. Monoclonal rabbit anti-ASC (EPR10402(B)) was purchased from Abcam. Monoclonal mouse anti-*β*-actin (A5316) was purchased from Sigma. Goat anti-rabbit (AP132P) and goat anti-mouse second antibodies (AP124P) were purchased from Merck.

The microplate reader, electrophoresis apparatus, film transfer apparatus, and gel imaging system were obtained from Bio-Rad (USA), the LEICADNLB2 binocular microscope was obtained from LEICA, the Shandon325 paraffin slicing machine was obtained from British Shandon Company, and the fall-automatic biochemical analyzer was obtained from Bio-Rad (USA).

### 2.2. Animal Model

Male Sprague-Dawley rats (5–6 weeks, 150 ± 10 g) were purchased from Hunan SJA Laboratory Animal Co. Ltd. and acclimatized for 7 days. Rats were kept at a controlled ambient temperature (24 ± 2°C) and relative humidity (60 ± 10%) under a 12/12 h light/dark cycle; water was available ad libitum. This study was reviewed and approved by the Animal Experiment Ethics Committee of Hunan University of Chinese Medicine and carried out in accordance with their recommendations.

### 2.3. JZLGD

JZLGD is composed of *Alisma orientale* (Zexie) (10 g), Cassis Tora (Juemingzi) (30 g), *Salvia miltiorrhiza* (Danshen) (10 g), Curcumae Radix (Yujin) (10 g), Sargassum (Haizao) (30 g), and Lotus Leaves (Heye) (10 g). All components were purchased from the First Hospital of Hunan University of Chinese Medicine, soaked in eight volumes of water for 30 min, boiled for 1 h, and filtered. Six volumes of water were added, and samples were boiled for 1 h and filtered. We mixed the constituents and concentrated the mixture to 2 g/ml using a rotary evaporator at 65°C and stored it at 4°C. Before use, it was diluted to the required concentration with normal saline.

### 2.4. Model Establishment

According to a previous study, the rats received a high-fat diet (HFD) (40 kcal% fat, 20 kcal% sucrose, and 2% cholesterol) for 12 weeks to develop NAFLD. The HFD was provided by Research Diets, Inc. (batch number: D09100301).

### 2.5. Experimental Design

Sprague-Dawley rats were randomly divided into five groups: the normal control group, the NAFLD model group, and three JZLGD-treated NAFLD groups (receiving 2.3, 4.6, and 9.2 g/kg of body weight, respectively). Rats in the normal control group were fed a normal diet (ND, 0.275 ppm cholesterol, 5.1% fat, 23.5% protein, and 50.3% carbohydrate), while the rats in the other groups received an HFD during the experimentation period (12 weeks + 6 weeks). After 12 weeks, rats in the JZLGD-treated NAFLD groups were administered different doses of JZLGD by oral gavage once daily for another period of 6 weeks; rats in the other two groups were given the same dosage of normal saline. Food intake was monitored daily, and body weight was measured weekly. After the intervention, the rats were euthanized by peritoneal injection of 1% sodium pentobarbital (40 mg/kg body weight). The blood and liver from each rat were rapidly removed for further studies.

### 2.6. Liver Index

After 18 weeks, the body weight and liver weight were measured, and the following formula was used to determine the liver index: liver index = (liver weight/body weight) × 100%.

### 2.7. Histopathology

For histopathological analysis, we collected the same liver tissue segment from each rat. Tissue segments were washed with ice-cold saline once or twice and kept in 4% paraformaldehyde. Tissues were cut into slices with a thickness of 4 *μ*m and stained with hematoxylin–eosin (H&E). Three practiced pathologists who were blinded to the study design performed all histopathological examinations. The ballooning degeneration score and the NAFLD activity score (NAS) were recorded. The scoring criteria were in accordance with the guidelines of the National Institutes of Health Clinical Research Network on nonalcoholic steatohepatitis.

### 2.8. Biochemical Analysis

The blood samples were centrifuged at 3000 rpm for 10 min to isolate the serum. We used a fall-automatic biochemical analyzer to evaluate the activity of liver enzymes in serum (alanine transaminase enzyme (ALT) and aspartate transaminase (AST)) and the serum levels of the triglyceride (TG), total cholesterol (TC), low-density lipoprotein (LDL), and free fatty acid (FFA).

### 2.9. Assessment of Serum IL-1*β*, IL-18, TNF-*α*, and IL-6 Levels

Blood was obtained from the abdominal aorta and centrifuged to obtain the serum. The serum levels of IL-1*β*, IL-18, tumor necrosis factor-*α* (TNF-*α*), and IL-6 were determined by ELISA according to the manufacturer's instructions.

### 2.10. Serum LPS Levels

Blood was obtained from the abdominal vein, placed at room temperature for 30 min, and centrifuged to isolate the serum. Serum LPS levels were measured with a kit according to the manufacturer's instructions. Firstly, samples were added to antibody-coated enzyme-labeled wells, and then, labeled antigens were added. The mixture was incubated at room temperature for 1 h. Afterwards, the plate was washed with PBST three times, avidin-HRP was added, and plates were incubated at 37°C for 1 h. The absorbance was measured at 450 nm. A standard curve was drawn to calculate the concentration of LPS.

### 2.11. Western Blot Analysis

We randomly selected five mice from each group, and 100 mg of the same segment of liver tissue was collected. Then, 1 ml RIPA buffer containing protease and phosphatase inhibitor cocktail was added. After homogenization on ice with a glass homogenizer for 30 min, the homogenate was centrifuged to obtain the hepatic tissue proteins. The protein concentration was determined by BCA assay. For each group, 40 *μ*g of protein was taken for the following experiments. Protein samples were separated by SDS-PAGE and transferred to PVDF membranes. After blocking with 5% nonfat milk in TBST for 1 h, membranes were incubated at 4°C overnight with the following primary antibodies: anti-NLRP3 (1 : 1000), anti-apoptosis-associated speck-like protein containing a CARD (ASC) (1 : 1500), anti-caspase-1 (1 : 500), anti-caspase-11 (1 : 500), anti-GSDMD (1 : 1000), anti-GSDMD N-terminus (GSDMD-N) (1 : 1000), anti-IL-1*β* (1 : 1000), anti-IL-18 (1 : 1000), and anti-*β*-actin (1 : 5000). After washing the membranes with TBST, the membranes were incubated at room temperature with secondary antibodies for 1 h. The protein bands were visualized with Western Bright ECL. Finally, the Quantity One software was used for the quantitative analysis of the protein bands.

### 2.12. Statistical Analysis

The grouped data were analyzed with SPSS 22.0. Comparison of two groups was performed by two-tailed Student's *t*-test. Comparisons among three or more groups were performed with one-way ANOVA. Results are expressed as the mean ± standard deviation (SD). The GraphPad Prism 6.0 software was used for statistical evaluations. *P* < 0.05 was considered to indicate a statistically significant difference.

## 3. Results

### 3.1. JZLGD Reduced Liver Index and Serum Lipid Levels

**Figure 1:**
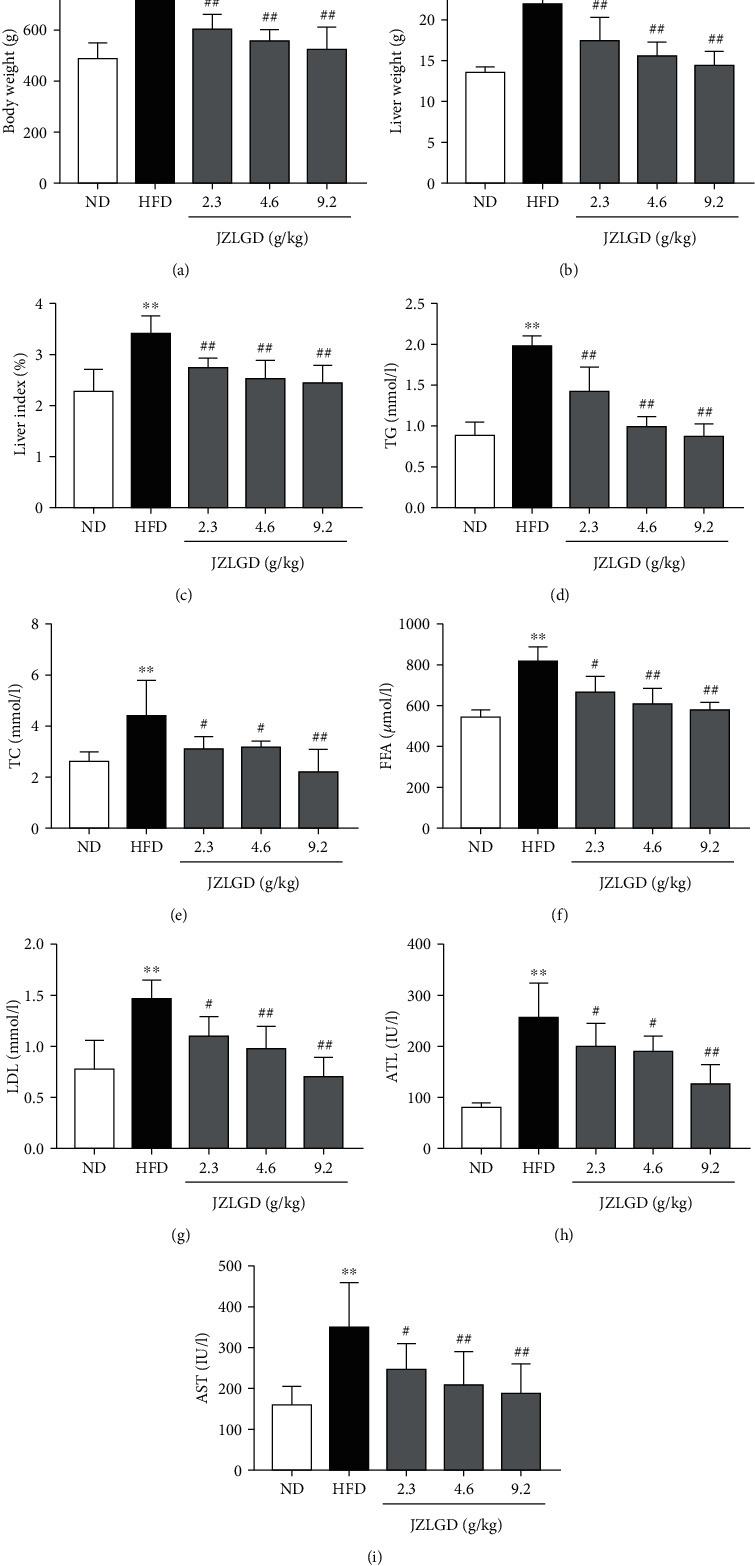
Rats fed with HFD for 12 weeks developed NAFLD. Next, rats were treated with 2.3, 4.6, or 9.2 g/kg JZLGD once a day for 6 weeks. (a–c) Body weight, liver weight, and liver index of NAFLD rats treated with JZLGD. (d–g) Serum TG, TC, FFA, and LDL levels as determined with a fall-automatic biochemical analyzer. (h, i) ALT and AST (enzymes related to liver injury) were detected to evaluate liver injury. Data are expressed as the mean ± SD (*n* = 10); ^∗∗^*P* < 0.01 vs. control; ^#^*P* < 0.05, ^##^*P* < 0.01 vs. HFD group.

To evaluate the therapeutic effects of JZLGD on NAFLD in HFD-fed rats, we measured the body weight, liver weight, liver index, serum lipid levels, and liver function parameters. The body weight, liver weight, and liver index were significantly higher in the NAFLD group than in the control group. After 6 weeks of JZLGD intervention (Figure 1), the body weight, liver weight, and liver index had improved significantly in the JZLGD-treated NAFLD groups, which proves that JZLGD blocks NAFLD development. Serum analysis results were in agreement with these observations. Serum TG, TC, FFA, and LDL levels were significantly higher in HFD-fed rats than in control rats. Compared to the HFD group, serum TG, TC, FFA, and LDL levels were greatly ameliorated in the JZLGD-treated NAFLD groups. High ALT and AST serum levels are associated with NAFLD progression; indeed, ALT and AST levels were higher in HFD rats than in control rats. Treatment with JZLGD reduced serum ALT and AST levels, indicating that JZLGD can protect rats from HFD-induced liver injury.

### 3.2. JZLGD Alleviated Pathological Morphology of Liver Tissue and Inflammation

**Figure 2:**
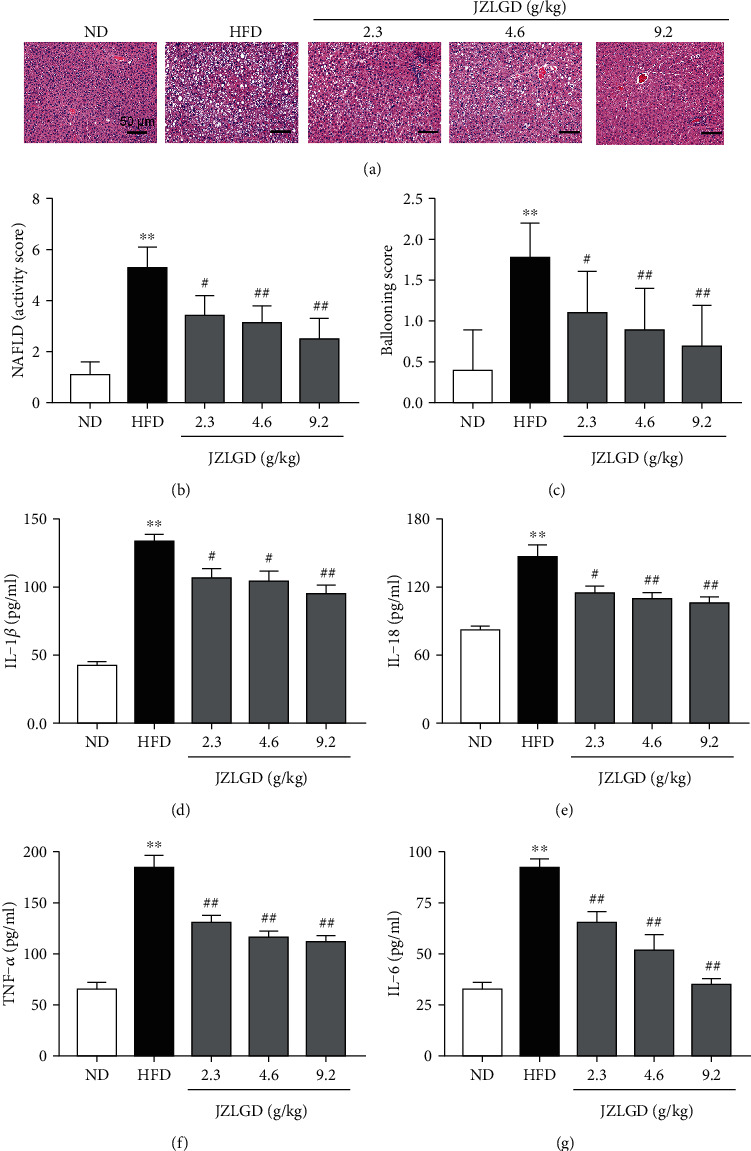
Effects of JZLGD on liver histology and hepatic inflammation-related parameters in HFD-fed NAFLD rats. (a) H&E staining (200x magnification) showed that inflammatory cell infiltration, balloon-like degeneration, and liver steatosis were obvious in the HFD group and inhibited in the JZLGD-treated NAFLD groups. Immediately afterwards, the NAFLD activity score (b) and (c) ballooning pathology score were recorded to evaluate the lesions better. (d–g) IL-1*β*, IL-18, TNF-*α*, and IL-6 serum levels were analyzed by ELISA. Data are expressed as the mean ± SD (*n* = 10); ^∗∗^*P* < 0.01 vs. control; ^#^*P* < 0.05, ^##^*P* < 0.01 vs. HFD group.

To further confirm the effects of JZLGD on the liver histopathology of NAFLD rats, we performed H&E staining, and the NAS and ballooning score were recorded. As shown in Figure 2, in the normal group, the hepatic lobules were intact, and the hepatocyte cords were arranged in an orderly fashion, with the central vein as the center in a radial arrangement. Hepatocytes were polygonal in shape, with large and round nuclei located centrally in the hepatocytes. A few lymphocytic infiltrates and no fibroproliferation were observed in the hepatic sinusoids and in the pooled areas. In the HFD group, the liver cells were disorderly arranged and accompanied by ballooning degeneration, infiltration of inflammatory cells, and marked hepatic steatosis, and hepatocytes were swollen and round, with large lipid droplets. Various degrees of steatosis were observed in the JZLGD-treated NAFLD groups, but the ballooning degeneration was reduced compared with that in the HFD group. These distinguishing features implied that HFD feeding can induce NAFLD and hepatic steatosis. Upon JZLGD treatment, hepatic steatosis and lipid accumulation gradually decreased. JZLGD treatment also effectively reduced the ballooning score and the NAS in NAFLD rats, in a concentration-dependent manner. This indicates that JZLGD has a positive protective effect on the liver.

We also detected the related inflammatory factors to assess the degree of hepatic inflammation in rats fed with HFD. Higher IL-1*β*, IL-18, TNF-*α*, and IL-6 levels were observed in the serum of HFD rats relative to the normal group. In JZLGD-treated NAFLD rats, IL-1*β*, IL-18, TNF-*α*, and IL-6 levels were significantly decreased, illustrating that JZLGD inhibits hepatocyte inflammation and prevents the progression of NAFLD.

### 3.3. JZLGD Inhibited the Expression of GSDMD and GSDMD-N

**Figure 3:**
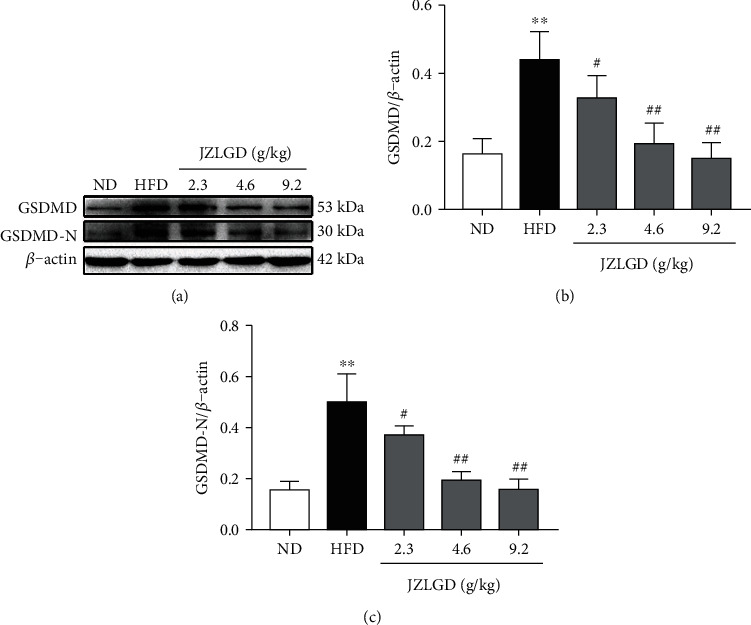
Effects of JZLGD on the levels of the pyroptosis executive protein GSDMD and GSDMD-N. (a) Western blot results. (b, c) Densitometric analysis of the relative band intensities of GSDMD and GSDMD-N. *β*-Actin was used as the internal control. Data are expressed as the mean ± SD (*n* = 10); ^∗∗^*P* < 0.01 vs. control; ^#^*P* < 0.05, ^##^*P* < 0.01 vs. HFD group.

The levels of the key protein GSDMD, which is the ultimate executor and the critical factor in pyroptosis, were measured by Western blot analysis. Moreover, the levels of its active form, GSDMD-N, in liver tissue were also evaluated. As shown in Figure 3, GSDMD and GSDMD-N levels were significantly higher in livers in the NAFLD group compared with the control group, confirming our conjecture that the occurrence of NAFLD is closely related to pyroptosis. Moreover, upon JZLGD treatment, the levels of GSDMD and GSDMD-N in the liver were significantly lower in the JZLGD-treated NAFLD groups. This effect was concentration-dependent. Therefore, JZLGD can effectively prevent the process of pyroptosis and protect hepatocytes.

### 3.4. JZLGD Suppressed the Levels of NLRP3, ASC, Caspase-1, IL-1*β*, and IL-18

**Figure 4:**
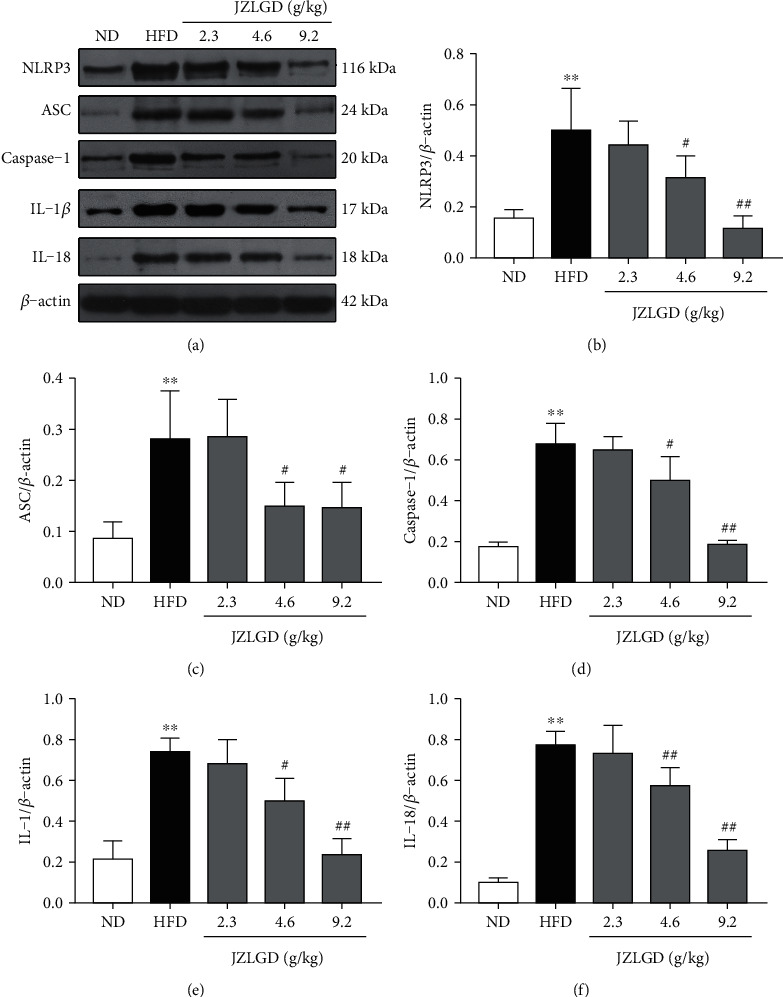
JZLGD reduced the expression levels of inflammasome-related proteins and proinflammatory factors involved in canonical pyroptosis signaling in HFD rats. (a) Representative blot and (b–f) quantitative analyses of (b) NLRP3, (c) ASC, (d) caspase-1, (e) IL-1*β*, and (f) IL-18 protein levels. Data are expressed as the mean ± SD (*n* = 10); ^∗∗^*P* < 0.01 vs. control; ^#^*P* < 0.05, ^##^*P* < 0.01 vs. HFD group.

To further explore the effects of JZLGD on the GSDMD-mediated canonical pyroptosis pathway, we measured the levels of NLRP3, caspase-1, and their adaptor protein ASC. As shown in Figure 4, the protein expression levels of NLRP3, caspase-1, and ASC in liver tissue were significantly higher in the HFD group compared with the normal group. Medium or high concentrations of JZLGD significantly decreased the NLRP3, caspase-1, and ASC protein levels. We also examined the levels of the proinflammatory factors IL-1*β* and IL-18, which act downstream of the pyroptosis pathway. The IL-1*β* and IL-18 protein levels of HFD rats were higher than those of normal rats, and JZLGD successfully suppressed the expression of IL-1*β* and IL-18. These results are consistent with our previous study.

### 3.5. JZLGD Suppressed the Expression of Caspase-11 and LPS

**Figure 5:**
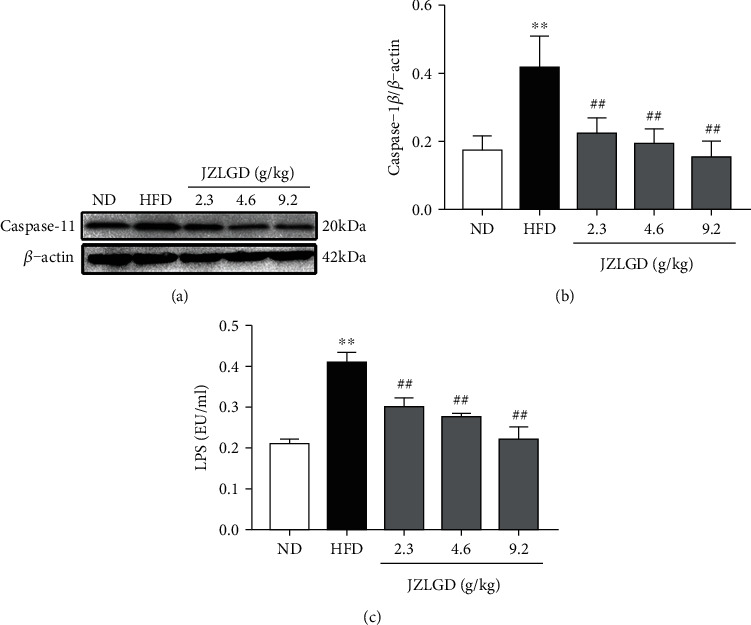
Effects of JZLGD on hepatic caspase-11 levels and serum LPS levels of HFD rats. (a) Representative immunoblots for caspase-11. (b) Densitometric analysis of caspase-11 expression. *β*-Actin was used as the internal control. (c) Serum LPS levels in NAFLD rats. Data are expressed as the mean ± SD (*n* = 5); ^∗∗^*P* < 0.01 vs. control; ^##^*P* < 0.01 vs. HFD group.

We also measured the levels of caspase-11 and LPS, which are related to the GSDMD-mediated nonclassical NAFLD pathway. As shown in Figure 5, the protein expression of caspase-11 in the liver tissue and the serum LPS levels were both significantly higher in the HFD group compared to the normal group. This suggests that the GSDMD-mediated nonclassical pathway also plays an important role in the development of NAFLD. Upon JZLGD treatment, hepatic caspase-11 levels and serum LPS levels were significantly reduced.

## 4. Discussion

NAFLD is rapidly developing into the main cause of liver cancer and liver cirrhosis in the world, yet there is no approved treatment drug [18]. Contrarily, JZLGD is a multi-ingredient pharmacon that affects multiple pathways, and its therapeutic effects for NAFLD treatment have been well documented [19, 20]. In the present study, the mechanisms underlying the effects of JZLGD on NAFLD were explored using an HFD-induced NAFLD rat model.

HFD rats exhibited increased body weight, liver weight, and liver index; upon JZLGD treatment, these indicators were effectively improved. Besides, JZLGD regulated blood lipid levels. ALT and AST activities also tended to be lower in JZLGD-treated rats, which suggested that JZLGD contributes to the prevention and alleviation of NAFLD in HFD-fed rats. Subsequently, histological examinations showed that JZLGD markedly mitigated liver lipid droplets. Furthermore, the NAS and the ballooning score were decreased in JZLGD-treated HFD rats, suggesting that JZLGD can slow down lipid accumulation. All these findings illustrate that JZLGD indeed exerts beneficial therapeutic effects on NAFLD.

HFD markedly increased the expression of hepatic inflammatory factors such as IL-1*β*, IL-18, TNF-*α*, and IL-6. Treatment with JZLGD obviously decreased these levels compared to the NAFLD model group, which suggests JZLGD attenuates hepatocellular inflammation. As the core of the pathogenesis of NAFLD, the inflammatory response plays an important role in the occurrence and development of NAFLD [21, 22]. Therefore, we speculate that the protective effect of JZLGD against NAFLD was probably mediated by the anti-inflammatory response. Recent studies have shown that pyroptosis can be regarded as a potential therapeutic target, as an inflammation-related and controllable cell death method [23] accelerates the process of hepatic inflammation and fibrosis [24, 25]. Notably, pyroptosis is a key pathogenetic factor in the development of NAFLD [23, 26], and suppression of inflammasome-dependent GSDMD-mediated cell pyroptosis could attenuate hepatic injury in liver diseases [27]. Therefore, we focused on the influence of JZLGD on pyroptosis in this study. We first detected the major executor protein GSDMD, which is a generic substrate of inflammatory caspases. Its cleaved form GSDMD-N acts as a pyroptosis executor, which triggers pyroptosis and aggravates the release of proinflammatory factors like IL-1*β* and IL-18 through the GSDMD pores [24]. Furthermore, inhibition of the activation of GSDMD blocks the progression of NAFLD. Hence, GSDMD is considered as a potential target for the treatment of NAFLD [9, 28]. Here, our experimental data show that the protein expression of GSDMD and its activated form GSDMD-N were significantly increased in HFD-induced NAFLD rats, which was consistent with previous studies [9] and further verified that GSDMD plays an important role in the occurrence and development of NAFLD. However, the fact that JZLGD treatment reversed the HFD-induced increase in the GSDMD and GSDMD-N expression indicated that JZLGD could regulate the activation of GSDMD, so as to achieve the purpose of treating NAFLD.

**Figure 6:**
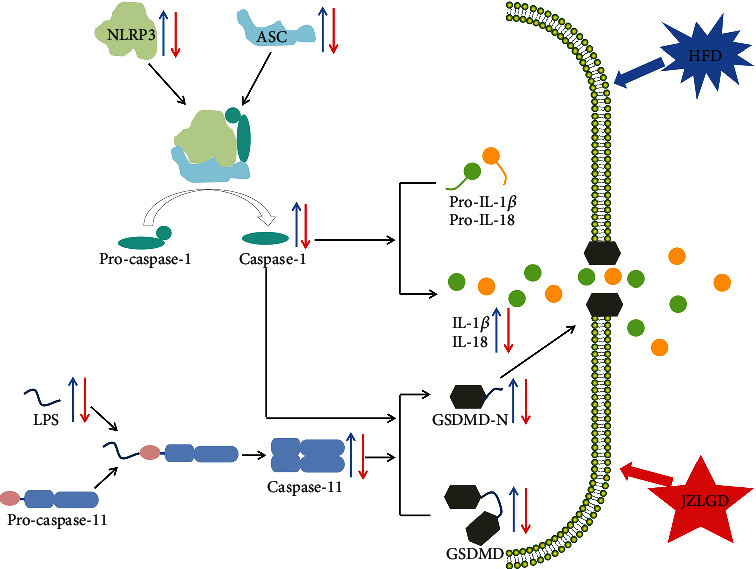
Graphical representation of the mechanism of action of GSDMD. During the process of pyroptosis, NLRP3 combines with ASC and the cysteine protease pro-caspase-1 to form inflammasomes. Inflammasomes can cleave pro-caspase-1, producing mature caspase-1. Mature caspase-1 can directly cleave pro-IL-1*β* and pro-IL-18 to generate the mature cytokines IL-1*β* and IL-18. In addition, LPS can directly bind to pro-caspase-11 and activate it. GSDMD is the executive molecule of pyroptosis and can be cleaved by activated caspase-1 and caspase-11. GSDMD-N then oligomerizes in the plasma membrane to form pores that increase membrane permeability, leading to IL-18 and IL-1*β* release, which mediates inflammation and pyroptosis. In the present study, 18 weeks of HFD led to some adverse consequences, such as weight gain, an increased liver index, higher blood lipid levels, pathological morphology, and inflammation of liver tissue in rats. It also led to an increase in NLRP3, ASC, caspase-1, LPS, caspase-11, GSDMD, GSDMD-N, IL-1*β*, and IL-18 levels. JZLGD treatment improved the above indicators and downregulated the expression levels of NLRP3, ASC, caspase-1, LPS, caspase-11, GSDMD, GSDMD-N, IL-1*β*, and IL-18.

As shown in Figure 6, GSDMD protein was cleaved to generate GSDMD-N, depending on the proinflammatory caspase-1 and caspase-4/5/11 via the canonical and noncanonical inflammasome signaling pathways [29]. Caspase-1 is the initiating factor of the canonical pyroptosis pathway, and its activation requires NLRP3 [30]. Furthermore, caspase-1 directly cleaves pro-IL-1*β* and pro-IL-18 to produce mature cytokines and controls their secretion to induce pyroptosis [31]. The expression levels of NLRP3, caspase-1, IL-1*β*, and IL-18 are key indicators for evaluating the activation of the NLRP3 inflammasome [32, 33]. Our results show that NLRP3, ASC, caspase-1, IL-1*β*, and IL-18 levels were elevated in HFD-induced NAFLD rats, which suggests that the NLRP3 inflammasome is also activated in NAFLD. JZLGD treatment decreased NLRP3, ASC, caspase-1, IL-1*β*, and IL-18 levels, suggesting that the mechanisms underlying the effects of JZLGD on HFD-induced NAFLD rats may be related to the NLRP3/caspase-1/GSDMD-mediated pyroptosis pathway.

Besides, in the noncanonical pyroptosis signaling pathway, GSDMD is cleaved by caspase-11, and caspase-11 is directly activated by LPS [34, 35]. Our results show that the levels of cleaved caspase-11 in liver tissue of HFD-induced NAFLD rats were significantly increased, while in the JZLGD treatment groups, we observed a significance decrease in caspase-11 levels, implying that JZLGD may regulate the activation of GSDMD through inhibiting the expression of caspase-11. It has previously been shown that disorder of the intestinal flora and damage of the intestinal mucosa can lead to the dissolution of Gram-negative bacilli in the intestinal tract, resulting in the release of LPS into the portal vein, which aggravates liver inflammation and accelerates NAFLD progression [36, 37]. Nicole et al. showed that HFD may induce changes or even disorders in the structure of intestinal flora, and Moreira et al.'s research showed that HFD can cause an imbalance in intestinal flora and lead to an increase in serum LPS levels [38, 39]. Moreover, some studies have revealed that JZLGD can regulate the intestinal flora and intervene with the progression of NAFLD [40]. Here, our data show that the LPS content in the hepatic portal vein of NAFLD rats was significantly increased, and JZLGD treatment could significantly reduce it. Based on the above results, we speculate that JZLGD may regulate serum LPS levels in NAFLD rats to prevent the activation of caspase-11 and GSDMD, thereby reducing the release of proinflammatory factors and attenuating the process of NAFLD. These findings illustrate that JZLGD treatment prevented steatosis, inflammation, and pyroptosis in HFD rats, which may be related to the LPS/caspase-11/GSDMD-mediated pyroptosis pathway.

## 5. Conclusions

In conclusion, our findings demonstrate that the canonical and noncanonical pyroptosis pathways are activated in HFD-induced NAFLD rats. JZLGD inhibited the NLRP3/caspase-1/GSDMD-mediated canonical pyroptosis pathway and the LPS/caspase-11/GSDMD-mediated noncanonical pyroptosis pathway, exerting beneficial effects in HFD-fed NAFLD rats. In future studies, the specific mechanism by which JZLGD regulates pyroptosis needs to be elucidated. For example, does JZLGD reduce serum LPS levels by changing intestinal permeability? Further investigations of the specific mechanisms of action of GSDMD might lead to the development of effective drugs to treat NAFLD.

## Data Availability

The data generated during this study are included in this article and results as shown in figures.
